# Anterolateral Papillary Muscle Rupture After Acute Myocardial Infarction Leading to Severe Mitral Regurgitation: A Case Report

**DOI:** 10.1002/ccr3.71551

**Published:** 2025-12-01

**Authors:** Zeyan Liu, Shou Zhou, Xuexiang Li, Qi Yang, Xiaodong Pan

**Affiliations:** ^1^ Department of Emergency Internal Medicine Second Affiliated Hospital of Anhui Medical University Hefei Anhui China

**Keywords:** acute myocardial infarction, heart failure, mitral regurgitation, papillary muscle rupture

## Abstract

Due to dual blood supply, ischemic mitral anterolateral papillary muscle rupture is less common than that of posteromedial papillary muscle and is rare in acute myocardial infarction without left main lesion. “Pseudo‐normalization” phenomenon of left ventricular ejection fraction may occur in the early stage, which is inconsistent with clinical symptoms.

## Graphical abstract

Mitral anterolateral papillary muscle rupture with partial valve body prolapse and severe mitral regurgitation after acute myocardial infarction (ultrasound schematic).

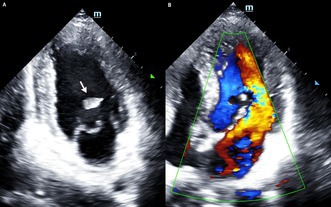

## 1 Introduction

Ischemic mitral papillary muscle rupture (MPMR) is a severe complication that may occur 2–7 days after acute myocardial infarction (AMI). However, its overall incidence is relatively low (approximately 0.04%–0.1%), which is much lower than that of other mechanical complications such as ventricular septal perforation or free wall rupture [1, 2]. The anterolateral papillary muscle (APM) is located near the apex on the anterior wall of the left ventricle. It is larger in size than the posteromedial papillary muscle (PPM) and usually consists of a single main body, which is connected to the lateral part of the anterior leaflet of the mitral valve through chorda tendinea. The APM has a dual blood supply, mainly from the left anterior descending branch (LAD)‐diagonal branch and the left circumflex branch (LCX)‐obtuse marginal branch (OM), while the PPM is supplied by a single vessel [3]. Therefore, in terms of pathogenesis, APM rupture is rarer in AMI patients. However, the recent registry (i.e., NCDR) showed that the incidence of APM rupture is increasing due to delayed reperfusion [4]. It often leads to more pronounced leaflet prolapse and flail motion, causing severe MR. This can severely disrupt the overall structure and contractile coordination of the left ventricle. During the compensatory phase of cardiac function, patients may show a falsely elevated left ventricular ejection fraction (LVEF), which can easily lead to clinical misjudgment. Once the decompensation phase is reached, the condition deteriorates rapidly. This is characterized by a sudden increase in cardiac volume load and a rapid decline in myocardial contractile function, resulting in severe hemodynamic disturbances. Early identification and accurate diagnosis are crucial to buy time and create conditions for surgical intervention, which is the key to successful treatment [5].

## 2 Case History

This study reports a case of a 73‐year‐old male patient. He had no history of cardiovascular disease, diabetes, or hypertension, but had a long‐term smoking history (over 30 years). The patient presented with sudden and persistent chest tightness for 3 days, which progressively worsened. He also experienced typical HF symptoms such as paroxysmal nocturnal dyspnea and orthopnea. Physical examination revealed that the patient was conscious, but had an acute illness appearance. He was orthopneic, and auscultation of both lungs revealed widespread crackles. His blood pressure was 90/74 mmHg, and his heart rate was 103 beats per minute. The cardiac dullness was enlarged, and a Grade III–IV holosystolic murmur was audible in the apical area. Bilateral lower extremity edema (3+) was also present.

## 3 Examination and Diagnosis

**FIGURE 1:**
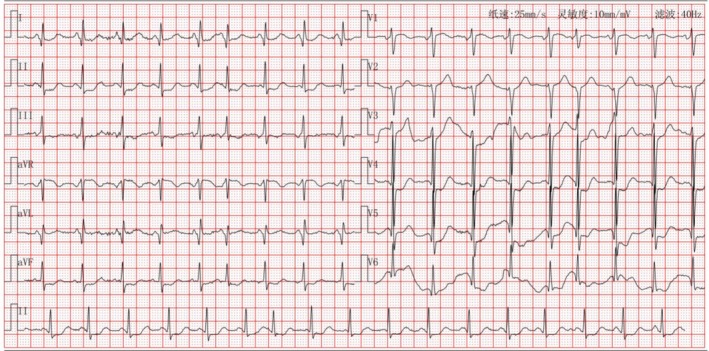
First ECG showed poor R‐wave increasing in the anterior wall and ST segment depression.

The initial electrocardiogram upon admission showed poor R‐wave progression in the anterior leads (Figure 1). Troponin I (CnTI) was 7.03 ng/mL (< 0.04 ng/mL), myoglobin was 269.1 ng/mL, CK‐MB was 12.29 ng/mL, and NT‐proBNP was 12059.0 pg/mL. Based on the clinical symptoms and physical examination findings, the diagnosis was considered as HF following AMI.

**FIGURE 2:**
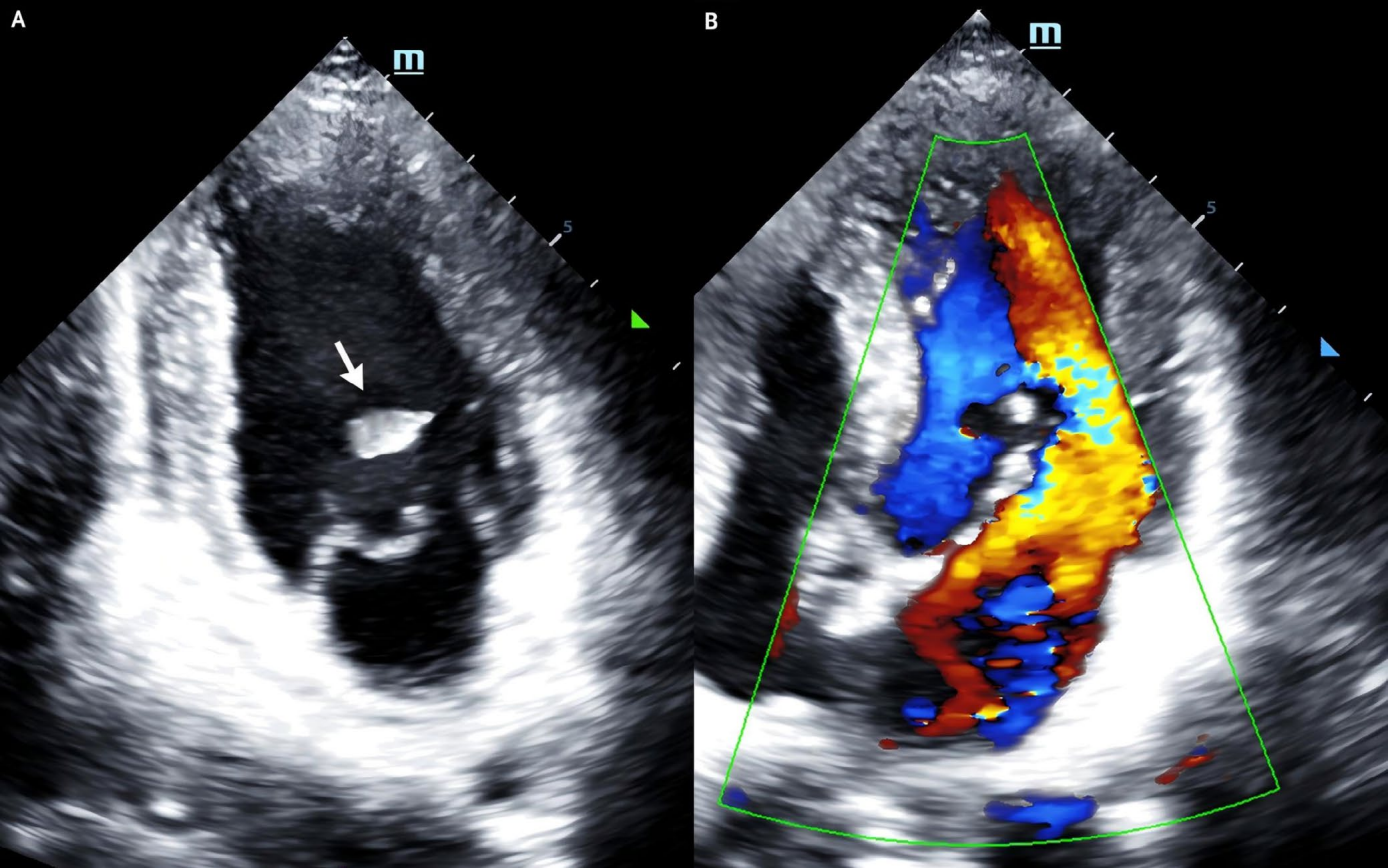
Transthoracic echocardiography showed mitral anterolateral papillary muscle rupture with partial valve body prolapse (A), severe mitral regurgitation (B).

**FIGURE 3:**
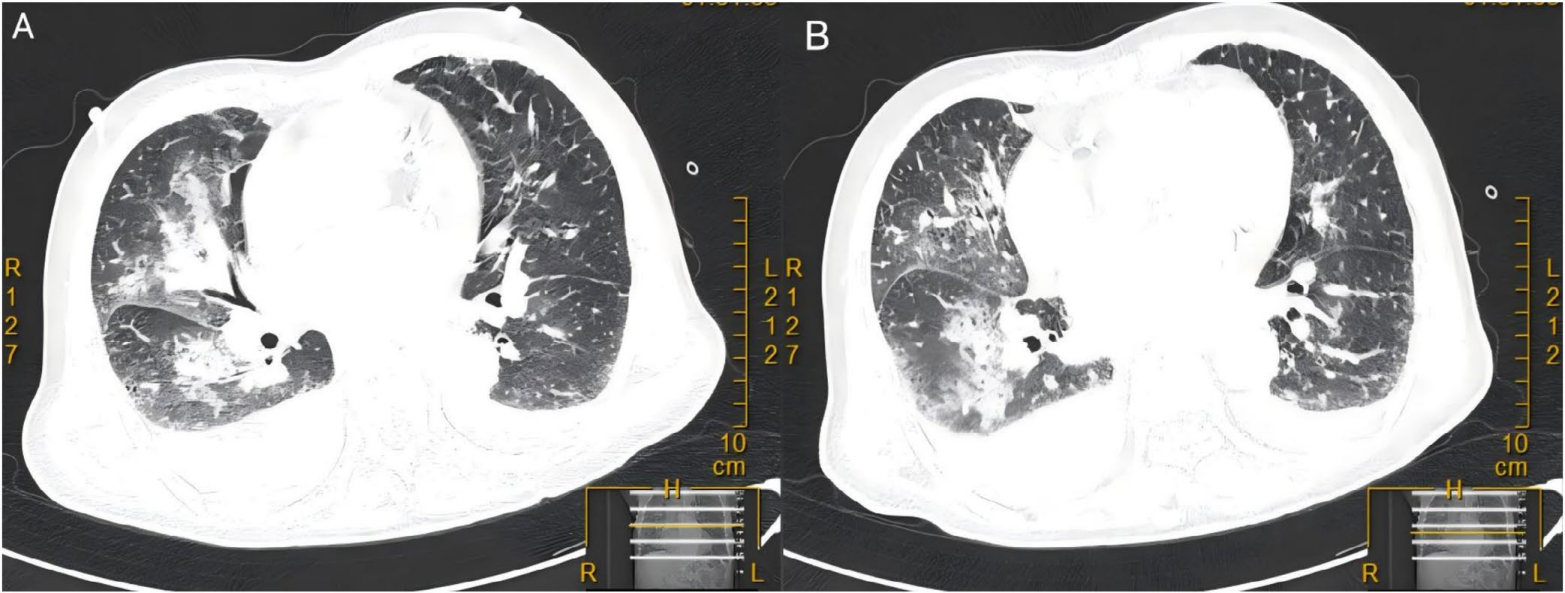
Chest CT showed bilateral pulmonary edema with pleural effusion (A and B).

Transthoracic echocardiography showed abnormal motion in the mid and lower segments of the left ventricular anterolateral wall, thickness of the interventricular septum and free wall is within the normal range, and the continuity is complete (no signs of rupture); no effusion was found in the pericardial cavity. Rupture of the APM of the mitral valve and significant prolapse of the anterior leaflet in the A1 and A2 regions during systole (with a maximum prolapse distance of 14 mm × 8 mm), along with characteristic flail motion, were noted. The main body of the APM was absent, with edematous changes at the stump, while the structure of the PPM remained intact. Ventricular remodeling was characterized by a relatively full left ventricle and a small right ventricle. Left ventricular end diastolic diameter (LVEDD) was 50 mm, and enlargement of the left atrium was 42 mm. Notably, the LVEF was maintained at 60% despite the papillary muscle rupture. Pulmonary artery pressure (PAP) was 27 mmHg (Figure 2A,B). Chest CT revealed bilateral pulmonary edema and pleural effusion (Figure 3A,B).

The detailed transthoracic echocardiographic image material is shown in the embedded Videos 1 and 2.

**VIDEO 1:** Echocardiography showed rupture of the anterolateral papillary muscle of the mitral valve, and significant prolapse of the anterior leaflet in the A1 and A2 regions during systole (with a maximum prolapse distance of 14 mm × 8 mm), along with characteristic flail motion. The main body of the anterolateral papillary muscle was absent, with edematous changes at the stump. Video content can be viewed at https://onlinelibrary.wiley.com/doi/10.1002/ccr3.71551.

**VIDEO 2:** Echocardiography showed severe mitral regurgitation, with regurgitant beams impinging on the atrial wall. Video content can be viewed at https://onlinelibrary.wiley.com/doi/10.1002/ccr3.71551.

**FIGURE 4:**
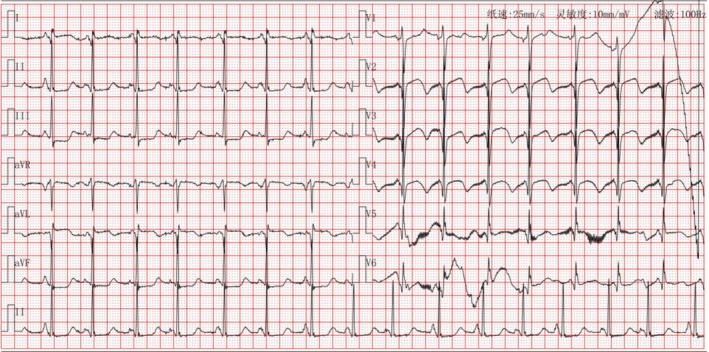
Second reexamination ECG showed an abnormal Q wave of the anterior wall with ST segment elevation.

**FIGURE 5:**
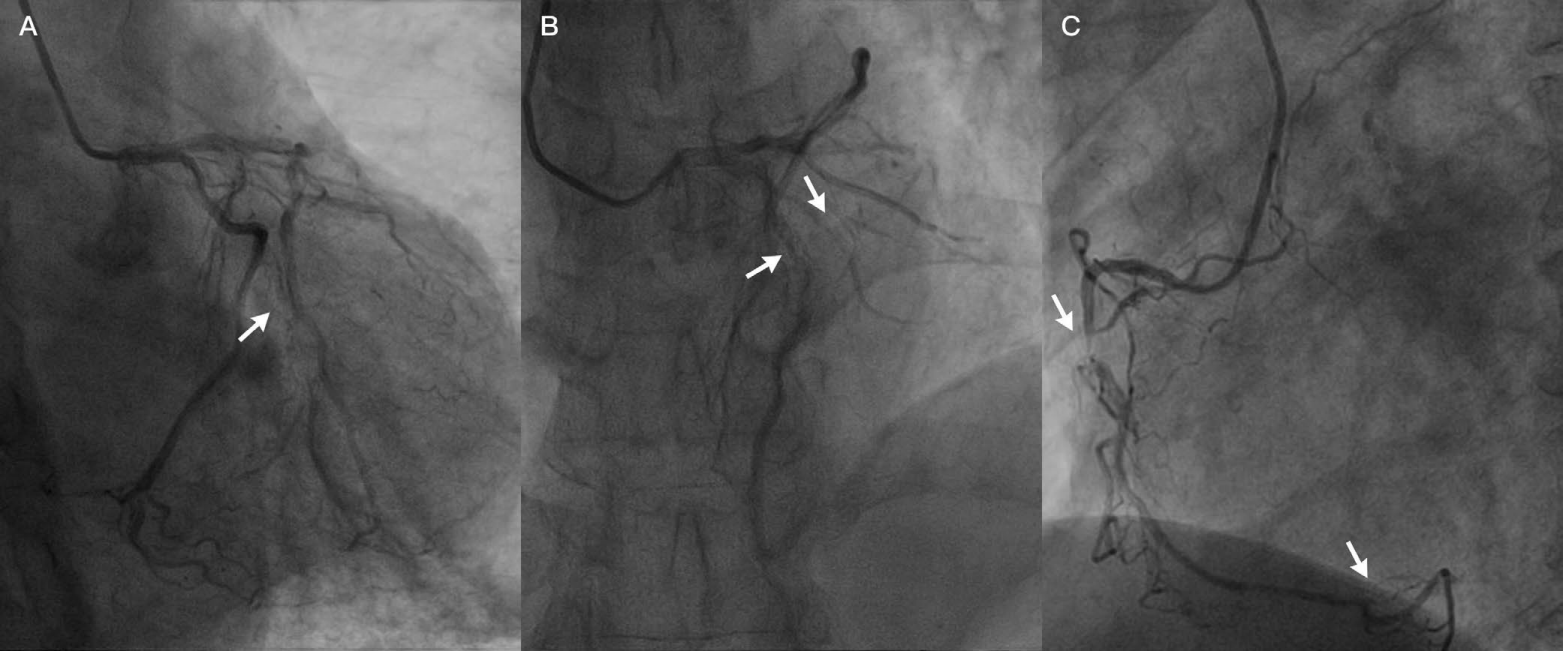
Coronary angiography showed chronic occlusion of the blunt border branch (A), severe stenosis of the anterior descending branch with thrombus shadow, severe stenosis of the diagonal branch, and significantly slowed blood flow in the diagonal branch (B), severe stenosis of the proximal right coronary artery and occlusion of the posterior branch (C).

Recheck high‐sensitivity Troponin I (Hs‐CnTI) was 6475.4 pg/mL (0.0–42.9 pg/mL). Serum *Treponema pallidum* antibody (−), antinuclear antibody spectrum (−). The second ECG after admission showed pathological Q waves in the anterior leads with persistent ST segment elevation (Figure 4). Further coronary angiography (CAG) revealed the following: There was a 60% luminal stenosis in the proximal and middle segments of the LAD with intraluminal thrombus formation, and 75% segmental stenosis of the middle diagonal branch with significantly slowed blood flow velocity, TIMI Grade I–II. The LCX showed diffuse irregularity of the vessel wall with chronic total occlusion in the proximal segment of the obtuse marginal branch, with a few collateral circulations originating from the LCX remote segment visible (Rentrop Grade 1). There was a 90% severe stenosis in the middle segment of the right coronary artery (RCA) with complete occlusion of the posterolateral branch, while the posterior descending artery had relatively preserved flow (Figure 5A–C).

Based on the examination results, the case was ultimately diagnosed as acute ST elevation myocardial infarction with Killip Class III–IV, complicated by rupture of the APM of the mitral valve, resulting in severe MR, holosystolic murmur, and acute heart failure with pulmonary edema. Preliminary analysis indicated that the cause of AMI in this patient was coronary atherosclerotic plaque rupture with acute thrombosis; the causes of HF after AMI were as follows: First, rupture of the mitral anterolateral papillary muscle rupture (MAPMR) led to severe MR. Second, myocardial ischemia and necrosis after AMI caused a decline in cardiac function. The cause of MAPMR was chronic total occlusion of the OM branch combined with plaque rupture and thrombus formation in the proximal and middle segments of the LAD, leading to a sharp reduction in blood flow in the diagonal branch. As the OM branch and the diagonal branch are the supplying vessels for the APM, the simultaneous severe lesions in these vessels were the main reasons for the rupture.

## 4 Treatment

In the first phase (preoperative treatment phase), a comprehensive conservative treatment regimen was employed to systematically manage HF resulting from AMI complicated by APMR. Noninvasive mechanical ventilation was used to assist ventilation, reduce preload, and provide positive pressure to aid cardiac contraction. Simultaneously, multitarget pharmacotherapy was implemented, including dual antiplatelet therapy with aspirin (0.1 qd) and ticagrelor (90 mg bid) to prevent thrombus progression, rosuvastatin (20 mg qn) for plaque stabilization, and a combination of furosemide (20 mg qd), spironolactone (20 mg bid), and dapagliflozin (10 mg qd) to reduce cardiac preload. Low molecular weight heparin calcium (5000 U qd) was used for anticoagulation. Within 20 h after admission, morphine hydrochloride was administered intravenously twice (5 mg each time, with a 6‐h interval) to induce sedation, reduce respiratory work, and dilate blood vessels. Based on daily clinical assessments, the use of diuretics (torsemide) and inotropic agents (dopamine, levosimendan) was dynamically adjusted. After 17 days of intensive treatment, the HF of the patient significantly improved, and he was successfully weaned off ventilator support. NT‐proBNP levels decreased to 2224.0 pg/mL, and Hs‐CnTI returned to 33.5 pg/mL (0.0–42.9 pg/mL).

**FIGURE 6:**
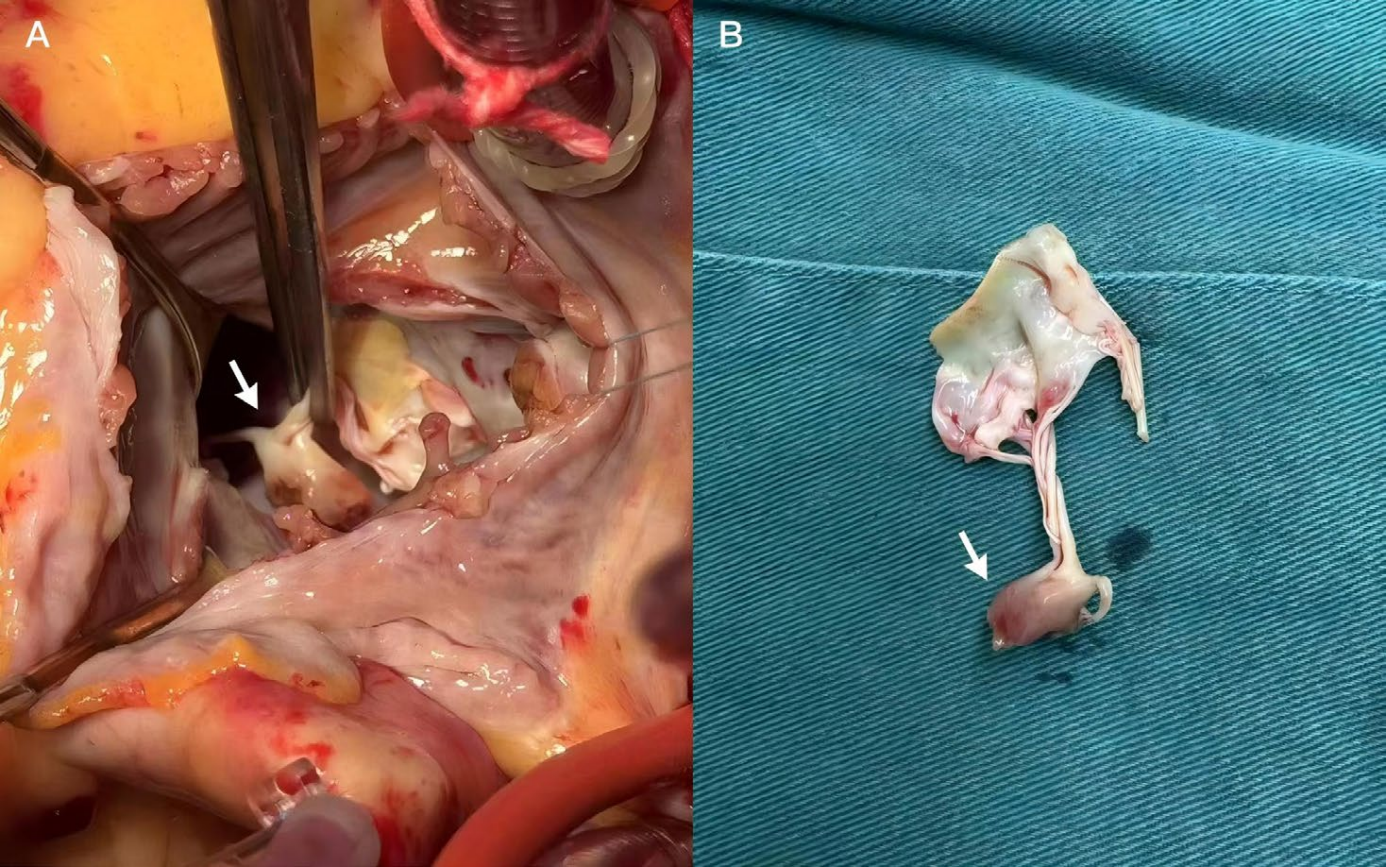
Surgically confirmed mitral anterolateral papillary muscle rupture (A and B).

**FIGURE 7:**
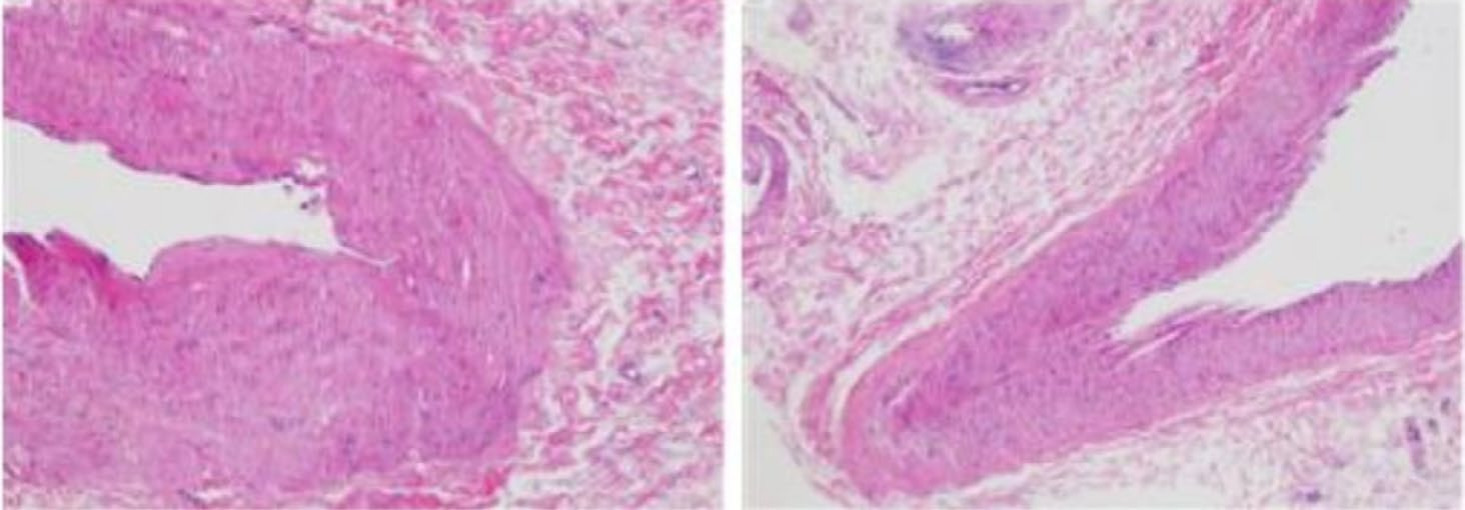
Postoperative pathological findings showed local ischemic necrosis changes.

In the second phase (operative treatment phase), the patient underwent mitral valve replacement with a mechanical valve and coronary artery bypass grafting under general anesthesia on the 18th day of admission after completing the preoperative evaluation. The surgery further confirmed APMR (Figure 6A,B), and the postoperative pathology revealed local ischemic necrosis changes (Figure 7).

In the third phase (postoperative treatment phase), the patient developed complications including low cardiac output syndrome, pulmonary infection, and acute kidney injury during the recovery process. On the basis of the original treatment, comprehensive interventions were implemented, including temporary pacemaker implantation and temporary intravenous dopamine infusion to stabilize the heart rate and improve low cardiac output, while also considering anti‐infection measures, renal protection, stabilization of the internal environment, nutritional support, and correction of coagulation function. After 27 days of systematic treatment, echocardiography showed that the mechanical valve at the mitral position was functioning essentially normally (PGmean: 5.7 mmHg, DVI: 2.37), with the left atrial anteroposterior diameter being 35 mm, LVEDD 45 mm, LVEF 47%, and PAP 27 mmHg. The patient was free of chest tightness and discomfort, could lie flat to sleep, had no crackles on lung auscultation, and no edema in the lower limbs, meeting the criteria for discharge.

## 5 Discussion

Studies have shown that the incidence of acute HF in the early phase after AMI is approximately 15%–30% [6]. The main cause is large‐area myocardial stunning or necrosis due to acute coronary artery occlusion, which subsequently leads to ventricular systolic and diastolic dysfunction. Results from trials such as VALIANT and GUSTO indicate that mechanical complications account for about 5%–10% of the etiology of HF after AMI [7, 8]. Among these, papillary muscle rupture, as a relatively rare mechanical complication, only represents 1%–3% of HF cases after AMI, with an overall incidence maintained at the relatively low level of 0.04%–0.5% [1, 2]. The characteristic manifestation of this complication is sudden severe mitral regurgitation, which often rapidly progresses to a state of cardiogenic shock. Surgical intervention is the optimal treatment approach. Statistics reveal that for patients who do not undergo surgical treatment, the 24‐h mortality rate is extremely high [9, 10]. However, due to the inherent risks of surgery and postoperative complications, the mortality rate after surgical intervention remains as high as 20%–40% [11].

The incidence of rupture of the APM after ACI is significantly lower than that of the PPM. This is mainly attributed to the dual blood supply (LCX‐OM and LAD‐diagonal branch) of the APM and its more robust conical structure, which endows it with greater mechanical stability. The reported incidence of its rupture may be < 0.1% [12]. However, APMR causes more severe clinical symptoms and higher death risks than posteromedial papillary muscle rupture (PPMR). The mechanisms are multifaceted. Anatomically, the APM mainly supports the key functional area of the anterior leaflet of the mitral valve. Its chorda tendinea are distributed centrally, and rupture easily leads to extensive uncontrollable prolapse of the valve leaflets. Meanwhile, due to its anatomical location near the apex, the eccentric regurgitation it generates has a more violent impact on the atrium. Pathologically, APM rupture is often accompanied by extensive anterior wall myocardial infarction involving both the LAD and LCX. This large‐area myocardial ischemia not only directly impairs ventricular systolic function, but also forms a vicious cycle with the secondary severe mitral regurgitation, accelerating the development of cardiogenic shock. In terms of treatment outcomes, compared with PPMR surgery, APMR surgery has relatively lower surgical difficulty, intraoperative mortality, and postoperative low cardiac output syndrome incidence, and a higher 1‐year survival rate [13, 14, 15]. Therefore, early and accurate identification to give patients the opportunity to undergo surgical intervention is the key to successful treatment of APMR.

The clinical significance of this case lies in its unique pattern of coronary artery disease. The patient did not present with typical left main disease or extensive anterior wall myocardial infarction due to simultaneous occlusion of the LAD and LCX, but rather had severe issues in both the OM and the diagonal branch. CAG revealed chronic total occlusion of the OM proximal segment, and the collateral circulation from the LCX distal segment is of poor quality, which has a very limited protective effect on APM. The diagonal branch itself had severe stenosis, which was further exacerbated by thrombus formation secondary to plaque rupture in the proximal LAD. This unique distribution of vascular lesions led to localized abnormal motion of the anterolateral wall, with the main driver of heart failure being severe mitral regurgitation caused by rupture of the APM. Factors influencing the delay in diagnosis of this patient were mainly due to the patient's initial echocardiographic assessment showing an LVEF of 60%, yet the symptoms and signs of HF were very pronounced (dyspnea, pulmonary edema, and lower limb edema), representing a typical compensatory phase of mitral regurgitation with overall cardiac contractile function still able to compensate. That is, the false elevation of LVEF in the early stage of MR is inconsistent with the clinical symptoms, which causes some misleading diagnosis. In addition, because the patient had severe heart failure after admission and did not have the conditions to complete CAG in emergency, the reason for APM could not be explored at the first time. However, the patient was hospitalized after completing ECG and point of care testing (POCT) in the emergency room, and there was no hospitalization delay. The treatment strategy in this case highlighted the importance of cardiac color Doppler ultrasound precise assessment. The treatment team accurately identified the main contradiction of HF and adopted a phased treatment strategy: first, to improve the patient's overall condition through standardized HF treatment, and then, to implement definitive surgery after the condition stabilized, ultimately achieving satisfactory therapeutic results.

## Author Contributions

**Zeyan Liu:** conceptualization, writing – original draft. **Shou Zhou:** resources, supervision, writing – review and editing. **Xuexiang Li:** writing – review and editing. **Qi Yang:** data curation, visualization. **Xiaodong Pan:** writing – review and editing.

## Funding

The authors have nothing to report.

## Consent

The patients who participated in the study signed an informed consent form at the time of admission, allowing the use of their clinical data and any relevant imaging materials for research and publication. According to the applicable ethical guidelines, the privacy protection of patients was ensured, and the manuscript did not contain any personally identifiable information.

## Conflicts of Interest

The authors declare no conflicts of interest.

## Data Availability

The data that support the findings of this study are available from the corresponding author upon reasonable request.
